# Current News

**Published:** 1893-10

**Authors:** 


### Current News.

### CHICAGO DENTAL SOCIETY.

At the annual meeting of the Chicago Dental Society, held Tuesday evening, April 4, 1893, the following officers were elected for the ensuing year:

President, J. W. Wassall; First Vice-President, J. H. Woolly; Second Vice-President, Garrett Newkirk; Recording Secretary, L. L. Davis; Corresponding Secretary, Geo. J. Dennis; Treasurer, E. D. Swain; Librarian, J. H. Smyser.

*Board of Directors.*—Edmund Noyes, J. G. Reid, Geo. H. Cushing.

*Board of Censors.*—C. R. E. Carpenter, D. C. Bacon, H. W. Sale.

Geo. J. Dennis,
*Corresponding Secretary.*

### DENTAL LAW IN THE TERRITORY OF NEW MEXICO.

This law, approved February 23, 1893, provides for the appointment of five practising dentists as a Board of Dental Examiners. This Board is to meet at least once a year.

Section 4 requires that all persons engaged in the practice of dentistry shall file an application for a certificate to continue in practice, and those who have been in practice for "one year next preceding the passage of the act" are entitled to receive a certificate without examination.

All unregistered persons will not be permitted to practise dentistry in the Territory until regularly examined.

Persons presenting a diploma from a college recognized as reputable by the National Association of Dental Examiners will be entitled to receive a certificate upon the payment of five dollars.

### SOUTHERN DENTAL ASSOCIATION.

This Association met at Kindergarten Hall, Chicago, August 11, President Dr. B. Holly Smith in the chair.

The Executive Committee reported that, under the unusual circumstances, it was deemed best that the Association should adjourn until the next annual meeting, the present officers to hold over until then.

The books of the secretary and treasurer were found correct, and it was suggested that the dues for this year be remitted.

Upon this latter point there was some discussion, but the president decided that a unanimous vote would make the action legal. Dr. McKellops then offered a resolution to remit the dues for this year. It was unanimously adopted.

Charges were made against a member resident in Rome, Ga., of having violated the code of ethics in advertising a patented plate. Drs. Gordon White, N. A. Williams, and M. W. Foster were appointed a committee to correspond with the party charged in the complaint, and instructed to report at the next annual meeting.

On motion, the officers were permitted to hold over until the next annual meeting.

The selection of the place of meeting was, on motion, left with the Executive Committee, with instructions to report within six months.

Adjourned.

---

### RECENT PATENTS.

Following is a list of recent patents, reported specially for the International Dental Journal:

Trade-Marks.—23,027.—Dental Remedies and Specifics. Milford L. Pine, Fabius, N. Y. Filed April 10, 1893. Essential feature the word "Nopain."

23,176.—Anæsthetic. Hale Dental Company, Boston, Mass. Filed May 5, 1893. Essential feature the word "Orinda."

23,179.—Tooth-Brushes. Florence Manufacturing Company, Florence, Mass., New York, N. Y., and Chicago, Ill. Filed May 15, 1893. Essential feature the words "Dental Plate."

33,067.—Dental Rubber. Edward J. McCormick, New York, N. Y. Filed April 25, 1893. Essential feature the word "Anaconda."

493,800.—Dental Plate. John R. Watson, Smithfield, Pa. Filed October 7, 1890.

493,843.—Dental Plate. Johannes A. A. Schoondermark, Leeuwarden, Netherlands. Filed July 5, 1892.

